# Effect of Bicalutamide on the proliferation and invasion of human triple negative breast cancer MDA-MB-231 cells

**DOI:** 10.1097/MD.0000000000019822

**Published:** 2020-04-24

**Authors:** Yan Kong, Fanjie Qu, Xiaolin Yuan, Xin Yan, Weiwei Yu

**Affiliations:** a Department of Oncology, Dalian third People's Hospital; b Central Laboratory, Affiliated Zhongshan Hospital of Dalian University, Dalian, Liaoning, China.

**Keywords:** Bicalutamide, invasion, MDA-MB-231, proliferation, triple negative breast cancer cells

## Abstract

Previous studies have shown androgen receptor (AR) is associated with the occurrence, development, recurrence, metastasis, and prognosis of triple negative breast cancer (TNBC). More and more experts have noticed that AR signaling pathway plays an important role in the occurrence and development of TNBC. The purpose of this study is to detect the inhibitory efficacy and mechanism of Bicalutamide on the proliferation and invasion of TNBC cells.

MDA-MB-231 cells of human breast cancer cells were treated with 0, 25, 100 μmol/L of Bicalutamide, cell proliferation assay was performed to assess cell proliferation viability by 3-(4,5-dimethyl-2-thiazolyl)-2,5-diphenyl-2-H-tetrazolium bromide, Thiazolyl Blue Tetrazolium Bromide assay and cell invasion was evaluated by Transwell assay. Meanwhile, flow cytometric analysis and western blotting were performed to investigate the mechanism of Bicalutamide on the proliferation and invasion of MDA-MB-231 cells.

Bicalutamide could efficiently inhibit the proliferation and invasion of MDA-MB-231 cells in a dose-dependent manner. In addition, Bicalutamide could significantly induce the cell cycle arrest at G0/G1 phase and decrease the protein expression of AR, cyclin D1, matrix metalloprotease-2 (MMP-2), and matrix metalloprotease-9 (MMP-9).

The present study indicated the Bicalutamide inhibited the proliferation and invasion process of triple negative breast cancer cells by targeting AR signaling pathway and down-regulating MMP-2/-9 protein expression levels.

## 1 Introduction

Breast cancer is one of the most common female malignant tumors in the world. Triple negative breast cancer (TNBC) is a group of breast cancer with special biological behavior and clinical pathological characteristics, which is characterized by high histological grade, strong invasion, poor prognosis compared with other subtypes of breast cancer, and lack of corresponding targeted treatment.

Although many therapeutic methods for TNBC have been developed, recurrence and metastasis still remain. The treatment for TNBC is an urgent clinical problem. Studies have found that androgen receptor (AR) is related to the occurrence, development, recurrence, metastasis, and prognosis of triple-negative breast cancer, and AR signaling pathway plays an important role in the occurrence and development of TNBC.^[1–3]^ More and more experts and scholars have noticed that AR may be a new therapeutic target for patients with breast cancer, especially for patients with ER–/PR–/AR+ subtype,^[4,5]^ and inhibition of AR pathway may be effective for the treatment of TNBC. The purpose of this study was to evaluate the possibility of clinical application of Bicalutamide by investigating the effect of androgen inhibitor Bicalutamide on the proliferation and invasion functions of TNBC cells, so as to provide the experimental basis for its clinical application.

## 2 Materials and methods

### 2.1 Materials

This study was approved by the Dalian municipal health and family planning commission and supported by the Natural Science Foundation of Dalian City, Project No. 1611037. Highly metastatic human breast cancer lines (MDA-MB-231) were obtained from the American Type Culture Collection. DMEM medium was purchased from Gibco; Trypsin, MTT, and DMSO were purchased from Sigma Chemical Company (Beijing, China). Transwell chamber is a product of BD company (New Jersey, US); Bicalutamide was purchased from Astrazeneca: Shanghai, China; Rabbit monoclonal against AR, Cyclin D1, matrix metallo proteinase 2 (MMP2), and matrix metallo proteinase 9 (MMP9) were purchased from American CST Corporation: MA 01923 Global Offices.

### 2.2 Cell culture

MDA-MB-231 cells were cultured in DMEM medium (Gibco, Carlsbad, CA) supplemented with 10% (v/v) fetal bovine serum (FBS) (Gibco-BRL, Grand Island, NY), penicillin (100 U/mL), and streptomycin (100 μg/mL) at 37 °C in a humidified atmosphere of 95% air and 5% CO_2_. The state of cell growth was observed under an inverted microscope.

### 2.3 Cell proliferation assay

Cell proliferation assay was performed to assess cell proliferation viability by MTT assay. The experiment was divided into solvent control group and drug intervention group. DMSO with 0.1% final concentration was used as solvent control group, and the final concentration of drug intervention group was 25 and 100 mol/L, respectively. MDA-MB-231 cells in logarithmic growth phase were seeded in 96-well plates at a density of 4000 cells/well and treated with Bicalutamide with the indicated concentrations (0, 25, 100 μmol/L). After 24, 48, and 72 hours exposure, the culture medium was removed, 20 μL of MTT (0.5 mg/mL) was added to each well and incubated at 37 °C for 4 hours. Then MTT was removed, and 200 μL of dimethyl sulfoxide was added to dissolve the formazan crystals, then the absorption was determined at a wavelength of 570 nm.
 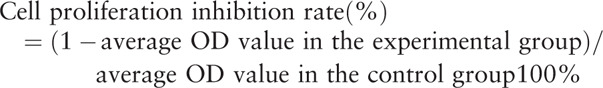


### 2.4 Transwell invasion assay

Transwell invasion assays were performed using MDA-MB-231 cells after Bicalutamide treatment. Cells were seeded in 6-well plates at a density of 5 × 10^5^ cells/well and incubated with serum-free medium containing Bicalutamide (0, 25, 100 μmol/L) for 48 hours. Cells were washed 3 times with PBS and digested into single cell suspension (1 × 10^5^ cells/mL) containing 1% BSA. Coated the top of the chamber with Matrigel (SKBR3: 30 μL serum-free medium, 1:7) before adding cell suspension. Cells on the upper surface of membrane were wiped off with cotton swab after incubating in the incubator at 37 °C for 48 hours. The migratory cells attaching to the outside bottom surface of the membrane were fixed with methanol and stained with 0.1% crystal violet for 15 to 20 minutes respectively. Cells on the bottom surface of the membrane filter were counted in 5 randomly selected visual fields under an inverted microscope at 100× magnification. Data were presented as the average number of cells adhering to the bottom surface.

### 2.5 Flow cytometry

Assessment of cell cycle and apoptosis by flow cytometry. MDA-MB-231 cells were treated with Bicalutamide at indicated concentrations for 48 hours. The cells were incubated in an incubator before they were harvested by centrifugation. Subsequently, the cells were fixed gently by 70% ethanol at 4 °C for overnight and were then re-suspended in PBS containing 10 μL PI (1 g /L). After incubation at 37 °C for 20 minutes, the Multicycle System was used for cell cycle analysis. Repeat 3 times for each set.

### 2.6 Western blotting

MDA-MB-231 cells were lysed in RIPA lysis buffer. Protein concentration was determined with bicinchonininc acid method. The amounts of lysates were resolved on sodium dodecyl-polyacrylamide gel electrophoresis (SDS-PAGE). Then, the proteins were transferred onto a polyvinylidene fluoride (PVDF) membrane. Subsequently, the membrane was blocked with 1× TBS containing 0.05% Tween-20 and 2% BSA at room temperature (25 °C) for 1 hour. Then, the membranes were incubated overnight at 4 °C with the respective primary antibodies (AR, Cyclin D1, MMP-2, MMP-9). The membranes were washed with 1× PBS and incubated with green fluorescence secondary antibodies in the dark for 1 hour at room temperature, and then the membranes were detected at 800 nm.
 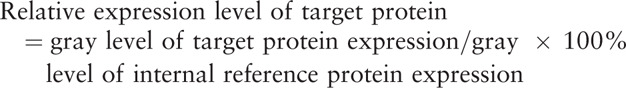


### 2.7 Statistical analysis

The experiments were conducted in triplicate, and SPSS 16.0 software (Armonk, NY, USA) was used for statistical analyses. The comparison between 2 groups was evaluated using Student *t* test. Multiple comparisons between the experimental groups and control groups were made using Tukey HSD when the probability for analysis of variance was statistically significant. The results were expressed as means ± SD. Values of *P* < .05 were considered statistically significant.

## 3 Results

### 3.1 The inhibitory effects of Bicalutamide on proliferation of MDA-MB-231 cells

To determine the anti-tumor activity of Bicalutamide in breast cancer MDA-MB-231cells, the cells were treated with various concentrations of bicalutamide (0, 25, 100 μmol/L) for 24 to 72 hours. After 24 hours exposure, the inhibition rates were 29.45% ± 1.23% and 45.31% ± 4.23%; after 48 hours exposure, the inhibition rates were 39.33% ± 1.23% and 62.31% ± 5.23%; after 72 hours exposure, the inhibition rates were 46.13% ± 2.63% and 74.31% ± 5.28%. Compared with the control group, cell inhibition rate significantly increased in Bicalutamide group (*P* < .05). The results showed that Bicalutamide decreased the proliferation of MDA-MB-231 cells significantly in a dose-dependent manner.

### 3.2 Effect of Bicalutamide on invasion of MDA-MB-231 cells

To determine the effect of Bicalutamide on invasion in MDA-MB-231 cells, we detected the effect by transwell migration and invasion assay.

After 48 hours exposure, as the Bicalutamide concentration was increased, the number of cells passing through the membrane was decreased and OD values were 0.561 ± 0.062, 0.318 ± 0.054, respectively. Compared with the control group, Bicalutamide treatment exhibited obvious inhibitory effects on the migration of MDA-MB-231 cells (*P* < .05). The results indicated that Bicalutamide played a vital role in inhibiting invasion and migration of MDA-MB-231 cells.

### 3.3 Effects of Bicalutamide on the cell cycle of MDA-MB-231 cells

After treatment with 25 and 100 mol/L Bicalutamide for 48 hours, the cells arrested in G0/G1 phase were obviously increased, meanwhile, the cells in S phase were obviously decreased in the Bicalutamide group compared with the control group (*P* < .05). (Table 1).

**Table 1:**
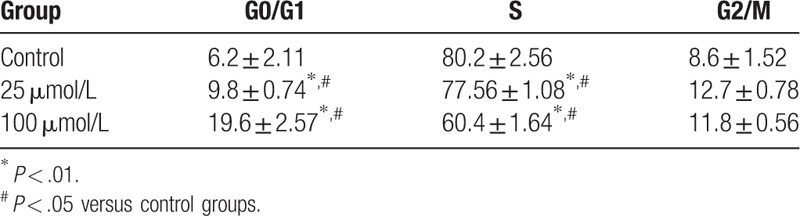
Effects of Bicalutamide on the cell cycle of MDA-MB-231 cells (% *x* ± *s*).

### 3.4 Western blot analysis

**Figure 1:**
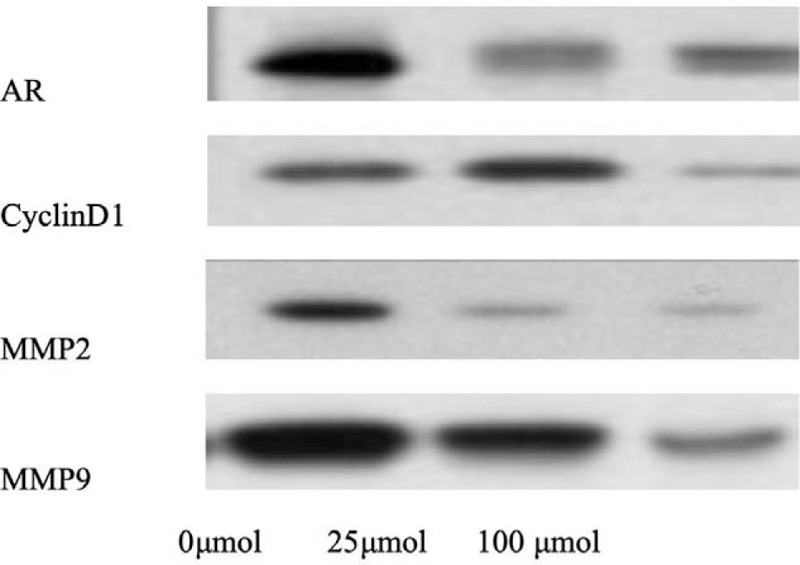
Bicalutamide affects the protein levels of AR, CyclinD1, MMP2, and MMP9 in MDA-MB-231 cells. Western blotting was utilized to determine the expressions of associated proteins, Bicalutamide could significantly decrease the protein expression of AR, CyclinD1, MMP2, and MMP9 in a dose dependent manner.

To further clarify the mechanism of Bicalutamide on the proliferation and invasion of triple negative breast cancer cells, we utilized western blotting to determine the expressions of associated proteins. Data showed that Bicalutamide could significantly decrease the protein expression of AR, CyclinD1, MMP2, and MMP9 in a dose-dependent manner (Fig. 1).

## 4 Discussion

Triple negative breast cancer (TNBC) is a group of breast cancer with special biological behavior and clinical pathological characteristics, which is characterized by high histological grade, strong invasion, poor prognosis compared with other subtypes of breast cancer, and lack of corresponding targeted treatment.

TNBC treatment is an urgent clinical problem. The important role of androgen and its receptor signaling pathway in the occurrence and development of prostate cancer has been confirmed. Bicalutamide, which plays a role by inhibiting androgen and its receptor pathway, has been applied in the clinical treatment of prostate cancer and achieved good efficacy.

In recent years, studies have found that the expression of AR is positive in >two-thirds of the triple negative breast cancer patients,^[6,7]^ the abnormal expression of androgen receptors in breast cancer tissue may be associated with invasion and migration of breast cancer,^[8]^ and hormone therapy can significantly inhibit the proliferation of cancer cells, meanwhile, it can promote cancer cell apoptosis by blocking androgen receptors,^[8–11]^ all these studies prompt AR may as a potential target TNBC treatment.

Malignant proliferation is an important characteristic of tumor cells, and the proliferation of tumor cells is related to the overexpression of cyclin, and CyclinD1 is a key protein that regulates the transformation of cell cycle from G0/G1 to S phase.

This study showed that Bicalutamide could significantly inhibit the proliferation of trinegative breast cancer cells. Compared with the control group, cell inhibition rate significantly increased in the Bicalutamide group (*P* < .05). The results showed that Bicalutamide decreased the proliferation of MDA-MB-231 cells significantly in a dose-dependent manner.

After treatment with Bicalutamide for 48 hours, the cells arrested in G0/G1 phase were obviously increased, meanwhile, the cells in S phase were obviously decreased in the Bicalutamide group compared with the control group (*P* < .05), and the expression level of CyclinD1 protein decreased significantly. This study prompted that Bicalutamide could efficiently inhibit the proliferation of MDA-MB-231 cells in a dose-dependent manner and the mechanism may be related to the decrease of CyclinD1 level, the arrest of cell cycle in G0/G1 phase.

Matrix metalloproteinases (MMPs), a class of membrane bound proteins that degrade extracellular matrix, have been shown to be important players in tumor angiogenesis, growth, and metastasis. In the MMPs family, mmp-2 and mmp-9 are closely related to tumor invasion and metastasis.^[12,13]^

Multiple studies have confirmed that the high expression of mmp-2 and mmp-9 is closely related to the clinical stage and metastasis of breast cancer, and the increase of these 2 proteases is more obvious in patients with early metastatic breast cancer.^[14–16]^

This study showed that Bicalutamide treatment can exhibit obvious inhibitory effects on the migration of MDA-MB-231 cells (*P* < .05). The results indicated that Bicalutamide played a vital role in inhibiting invasion and migration of MDA-MB-231 cells. At the same time, the expressions of MMP2 and MMP9 proteins in trinegative breast cancer cells treated with Bicalutamide were significantly decreased, and the expression of AR protein was also significantly decreased.

This study indicated that Bicalutamide could inhibit the invasion of MDA-MB-231 cells in a dose-dependent manner with effectiveness, and the mechanism may be related to the inhibition of AR signaling pathway, and the down-regulation of mmp-2, mmp-9, and other related proteins.

In conclusion, this study preliminarily confirmed in vitro that Bicalutamide can inhibit the proliferation and invasion of triple-negative breast cancer by inhibiting the androgen receptor signaling pathway, which is related to the down-regulation of cyclinD1, mmp-2, and mmp-9 related proteins.

The results showed that antiandrogenic therapy had certain inhibitory effect on trinegative breast cancer, which provided a new theoretical basis for the clinical application of Bicalutamide in trinegative breast cancer.

## Author contributions

**Investigation:** Xiaolin Yuan.

**Methodology:** Xiaolin Yuan, Fanjie Qu, Yan Kong.

**Project administration:** Fanjie Qu.

**Resources:** Yan Kong, Fanjie Qu, Xiaolin Yuan, Xin Yan, Weiwei Yu.

**Software:** Xin Yan, Weiwei Yu.

**Supervision:** Fanjie Qu.

**Writing – original draft:** Yan Kong, Fanjie Qu.

**Writing – review & editing:** Fanjie Qu.
